# Telelactation Support in Emergency Situations: Experiences From the Recent Devastating Earthquakes in Turkey

**DOI:** 10.7759/cureus.79399

**Published:** 2025-02-21

**Authors:** Mine Basibuyuk, Nalan Karabayir, Gökçe Celep, Ferhat Karademir, Aybüke Kacir, Demet Bilgin, Övgü Büke, Özlem Öçal, Evrim Senkal, Merve Kula, Yesfa Sebnem Özbay, Özden Gönültas, Betül Ulukol, Figen Dagli

**Affiliations:** 1 Pediatrics and Child Health, İstanbul Medipol University, İstanbul, TUR; 2 Department of Pediatrics, İstanbul Medipol University, İstanbul, TUR; 3 Childhood Disease, Amasya Universty, Amasya, TUR; 4 Pediatrics, İstanbul Medipol University, İstanbul, TUR; 5 Pediatrics, Health Sciences University Bagcilar Research and Training Hospital, İstanbul, TUR; 6 Pediatrics, Suleyman Yalcın Training and Research Hospital, İstanbul, TUR; 7 Pediatrics, Bayrampaşa Training and Research Hospital, İstanbul, TUR; 8 Pediatrics, Haliç University, İstanbul, TUR; 9 Pediatrics, Esencan Hospital, İstanbul, TUR; 10 Pediatrics, Ankara University School of Medicine, Ankara, TUR; 11 Pediatrics, Gazi University Faculty of Medicine, Ankara, TUR

**Keywords:** baby care, breast milk, lactation consultant, natural baby care, tele medicine

## Abstract

Breastfeeding initiation within the first hour of birth, exclusive breastfeeding for six months, and continuation up to two years or beyond, even during emergencies, are generally recommended. However, challenges in breastfeeding support in emergencies often result in increased infant vulnerability and reliance on formula feeding. This retrospective study evaluates telelactation services provided by the "Disaster Area Parent Support Group" after the devastating February 2023 earthquakes in Turkey. Telelactation, a telemedicine-based breastfeeding counseling method, was implemented to address breastfeeding challenges in disaster-affected regions.

Between February and September 2023, 46 mothers received telelactation counseling. Most consultations addressed issues such as low milk supply, breast refusal, and relactation. Following an average of 9.11 consultations per mother over 16.69 weeks, exclusive breastfeeding rates increased significantly, with 32.56% of infants exclusively breastfed compared to 6.98% at admission. Additionally, successful relactation was achieved in 71.42% of cases among mothers attempting it. All mothers reported satisfaction with the support received.

This study highlights the critical role of telelactation in emergencies, particularly in regions with disrupted healthcare infrastructure. While not a substitute for in-person consultations, telelactation proved effective in resolving breastfeeding issues, improving infant feeding practices, and offering psychological support to mothers. The findings underscore the necessity of equipping healthcare providers with telemedicine skills to sustain breastfeeding during crises. Limitations include the small sample size and technological barriers in disaster zones. Future research should explore broader applications of telelactation in emergencies to enhance maternal and child health outcomes.

## Introduction

The World Health Organization (WHO) and UNICEF recommend initiation of breastfeeding within the first hour of birth, exclusive breastfeeding for the first six months, and continuing breastfeeding for two years and beyond under all circumstances, including emergencies [1,2]. Breast milk adapts to the biological, social, and psychological needs of both mothers and infants, making it a safe, nutritious, and easily accessible food that is critical for protecting infants against infections [3,4]. Challenges in the protection, promotion, and support of breastfeeding lead to a decline in breastfeeding rates, resulting in the loss of 823,000 babies annually and an estimated annual loss of $341.3 billion worldwide [5,6]. Infants and young children are the most vulnerable group in emergencies due to their special nutritional needs, immature immune systems, and dependency on caregivers [7]. Studies have shown that children in emergencies are at risk of severe malnutrition as well as increased mortality from infectious diseases such as pneumonia, diarrhea, and measles [8]. Key interventions for infant and young child feeding in emergencies include promoting and protecting breastfeeding, minimizing risks associated with formula use in non-breastfed infants, and supporting safe, nutritious, and age-appropriate complementary feeding and Infant and Young Child Feeding in Emergencies (IYCF-E) should therefore be an integral part of all emergency responses [9,10]. Due to limited fluid and calorie intake, stress, and fatigue during emergencies, mothers may perceive their milk supply as inadequate [11]. Intense stress experienced by mothers after disasters can suppress the milk ejection reflex and temporarily decrease the milk supply. Fortunately, this condition can be improved within a few days through frequent breastfeeding and proper support [7]. Additionally, hormones released during breastfeeding, like oxytocin and prolactin, help alleviate stress for both mothers and infants [3]. If breastfeeding is not possible, relactation, wet nursing, and donor human milk should be considered in this order. In emergencies, the Operational Guidance on Infant and Young Child Feeding in Emergencies (OG-IFE) recommends supporting relactation for non-breastfed infants and promoting a transition to exclusive breastfeeding for infants under six months. Breastfeeding support should be provided to these mothers and continued until breastfeeding is reestablished [12].

Emerging in the 2000s in many health services, telemedicine is becoming increasingly widespread and involves the “transfer of medical information between sites via electronic communications to improve a patient's clinical health status” [13]. It can be particularly used in situations where patients and healthcare professionals cannot meet face to face due to various reasons such as disasters, wars, epidemics, or transportation difficulties, and has become increasingly popular in all medical fields after the COVID-19 outbreak with high effectivity and satisfying results for the patients [14,15]. Providing counseling on breastfeeding in emergencies is critical for preserving and improving children's health [7,9]. Breastfeeding counseling conducted via telemedicine is referred to as "telelactation" and can be carried out through various technological communication methods such as video conferencing, video calls, and web-based messaging [15]. Online counseling can be provided through live sessions, known as "synchronous service," or through the transmission of prerecorded videos, known as "asynchronous service" [16]. Telelactation has the potential to improve access to professional breastfeeding assistance and has been shown to be effective in increasing exclusive breastfeeding rates at one and six months postpartum, especially for first-time mothers, those with high school or university education, and those living in urban areas [17-19]. The use of innovative technologies such as wearable sensors and digital data repositories has been reported to further increase the effectiveness of virtual breastfeeding support [20]. Two recent earthquakes of magnitude 7.7 and 7.6 affected 11 provinces in Turkey, leaving behind widespread devastation with 50,783 people killed and 107,204 injured. According to the Turkish Ministry of Health, more than 2,500 babies were born in the affected area in the first week after the earthquakes [21]. The aim of our study was to evaluate the impact of telelactation services provided by the “Disaster Area Parent Support Group” to parents in the affected area.

## Materials and methods

Study design

This retrospective study assessed the effectiveness of telelactation services delivered by the Disaster Area Parent Support Group between February 2023 and September 2023. Established on the Telegram platform in February 2023 by volunteer pediatricians, the support group aimed to provide guidance on breastfeeding and infant care to parents in disaster-affected regions of Turkey, with a particular focus on the 11 provinces severely impacted by the February 2023 earthquakes. The study specifically examined infants aged 0 to 24 months who received telelactation support through this initiative.

Telelactation service process

Telelactation support was provided by a pediatrician with expertise in breastfeeding counseling. Each consultation lasted approximately 15-20 minutes and focused on breastfeeding techniques, breast-related concerns (e.g., mastitis, cracked nipples), and strategies to enhance milk production. Communication was conducted primarily through video calls on Telegram, supplemented by text or image exchanges as needed. When necessary, mothers shared videos or images of their breastfeeding positions and challenges to facilitate more precise assessment and guidance.

To uphold privacy and confidentiality, all multimedia content shared by mothers was exchanged exclusively through one-on-one encrypted chats with the consulting pediatrician, rather than within a public group setting. Telegram’s end-to-end encryption ensured that only the involved healthcare provider had access to these materials. Additionally, no multimedia content was stored on external servers, and all data remained within the secure Telegram platform, reinforcing the protection of maternal and infant confidentiality.

Awareness of the Disaster Area Parent Support Group was disseminated through multiple channels, including social media campaigns, word-of-mouth referrals, and direct recommendations from healthcare professionals active in disaster-affected regions. Pediatricians and lactation consultants actively promoted the group to mothers in need of breastfeeding support within hospitals, shelters, and temporary healthcare facilities. Furthermore, collaboration with non-governmental organizations (NGOs) and humanitarian aid groups facilitated broader outreach, ensuring that affected families were informed of the available telelactation services.

Mothers primarily accessed the support group using their personal mobile devices. While no official distribution of mobile phones pre-installed with the Telegram application was conducted, volunteers provided guidance on installation and platform navigation as needed. In some instances, community representatives and healthcare workers played a pivotal role in assisting mothers with technological barriers, ensuring that those with limited digital literacy could effectively utilize the telelactation services. These measures were implemented to enhance accessibility and inclusivity, particularly for vulnerable populations with restricted access to conventional healthcare resources.

Data collection and recording

Data collected during the consultations were systematically documented using a standardized data collection form specifically developed for this study. The form was designed following a comprehensive review of existing data collection methodologies in lactation counseling and telehealth services, ensuring alignment with best practices in the field. Its content was informed by a critical evaluation of previous studies on breastfeeding counseling, WHO recommendations on breastfeeding support services, and established approaches to data collection in telelactation.

The recorded information encompassed the infant's age, gender, mode of delivery, province of residence, reason for consultation, current feeding method, frequency of consultations, follow-up duration, and clinical outcomes. Data were collected in a systematic and structured manner, incorporating input from both the pediatrician and the mother to enhance accuracy and reliability.

To ensure data security and confidentiality, all records were securely stored in an encrypted file accessible exclusively to authorized researchers. No data were stored on external servers, and access was strictly controlled to uphold participant privacy. These measures were implemented in accordance with standard data protection protocols, ensuring compliance with ethical and regulatory guidelines for safeguarding sensitive information.

Follow-up process

The follow-up period was structured to range between two to four weeks, depending on the clinical needs of the mother-infant dyad. Follow-up intervals were adjusted based on individual requirements, with a standard approach involving contact every one to three days. More frequent follow-ups were conducted in cases requiring intensive support, particularly for relactation attempts, insufficient milk supply, breastfeeding refusal, hyperlactation, mastitis, or infants transitioning to complementary feeding. These cases necessitated closer monitoring and additional guidance throughout the follow-up period.

One month after the completion of the follow-up period, parents were contacted via Telegram or cellular phone calls, depending on their accessibility and preference, for a final evaluation. During this process, a standardized questionnaire was administered to assess infant nutritional status and parental satisfaction with the telelactation services.

Infant weight measurements were conducted at the nearest healthcare facility while the infants remained with their mothers, ensuring the continuity of care. To maintain measurement accuracy, all weight assessments were performed using high-precision infant scales with a sensitivity of 10 grams, and infants were weighed completely undressed. The results were directly communicated to the mothers for continued monitoring. At no point during the study were infants separated from their mothers, and all assessments were conducted while preserving the mother-infant dyad.

To enhance data reliability and security, all follow-up records were systematically documented and securely stored in an encrypted file accessible only to authorized researchers, ensuring confidentiality and data integrity.

Final evaluation

One month after the completion of the follow-up period, parents were contacted for a final evaluation via Telegram voice calls or cellular phone calls, depending on their accessibility and preference. This flexible approach ensured that all participants could engage in the evaluation process regardless of their communication infrastructure.

A standardized questionnaire, specifically developed for this study, was used to assess infant nutritional status, parental satisfaction with the telelactation services, and the perceived effectiveness of the intervention.

Infant nutritional status was evaluated based on maternal self-reports of infant feeding practices, including exclusive breastfeeding status, breastfeeding frequency, the introduction of complementary foods where applicable, and any reported feeding challenges. Additionally, weight gain trends were monitored through healthcare facility-based infant weight measurements, which were systematically documented during the follow-up period.

Parental satisfaction was assessed through structured multiple-choice and open-ended questions evaluating the adequacy of breastfeeding support, accessibility and convenience of the telelactation service, clarity and comprehensibility of the guidance provided, and overall perception of the intervention’s effectiveness. Parents were also encouraged to provide qualitative feedback on their experiences and suggestions for improving the service.

To ensure data reliability, confidentiality, and compliance with ethical research standards, all responses were systematically recorded and securely stored in an encrypted, password-protected file accessible only to authorized researchers.

 Ethical approval

This study was approved by the Istanbul Medipol University Faculty of Medicine Ethics Committee on October 27, 2023 (Approval Number: -10840098-772.02-6875).

## Results

A total of 46 mother-infant dyads received telelactation counseling. Among them, three mothers (6.52%) sought complementary feeding recommendations, whereas 43 mothers (93.48%) participated in one-on-one online breastfeeding counseling. The mean age of the children was 4.08±5.52 months, ranging from 0 to 24 months. The age distribution was as follows: 30 infants (65.22%) were aged 0-<3 months, 10 infants (21.74%) were aged 3-<6 months, 4 infants (8.70%) were aged 6-<12 months, and 2 young children (4.35%) were aged 12-24 months.

Among children under two years of age, 25 (54.35%) were female, and 21 (45.65%) were male. In terms of birth mode, 25 children (54.35%) were born via spontaneous vaginal delivery, whereas 21 children (45.65%) were delivered via cesarean section (Table 1).

**Table 1:** Demographic Information

Variable	Mean ± SD (Min-Max) / n	Median / (%)
Infant age (months)	3.91 ± 5.46 (0–24)	2
Gender of the Infant		
Female	25	54.35%
Male	21	45.65%
Mode of delivery		
Spontaneous vaginal	25	54.35%
Cesarean section	21	45.65%
Number of consultations (n)	9.11 ± 2.95 (4–16)	9
Follow-up duration (weeks)	16.69 ± 5.98 (2–24)	16

**Figure 1:**
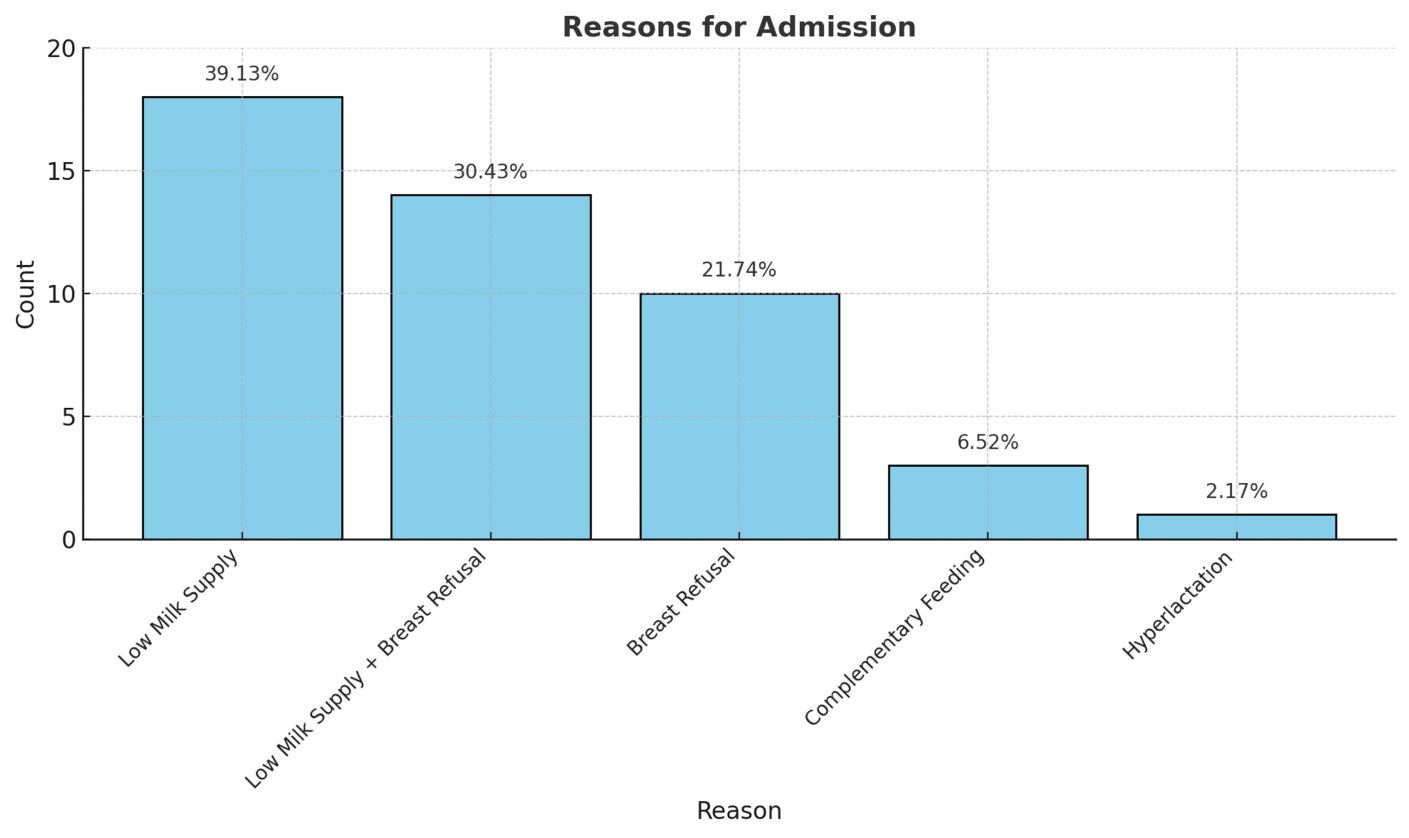
Reasons for Telelactation Consultation

Among the 43 mothers receiving breastfeeding counseling, the primary reasons for consultation included low milk supply (18 mothers, 39.13%), breast refusal (14 mothers, 30.43%), a combination of breast refusal and low milk supply (10 mothers, 21.74%), concerns regarding complementary feeding (3 mothers, 6.52%), and hyperlactation (1 mother, 2.17%) (Figure 1).

A total of 46 mothers sought telelactation support, with each receiving an individualized consultation plan tailored to their specific needs. The mean number of consultations per mother was 9.11±2.95 sessions (range: 4-16), with a follow-up period of 16.69±5.98 weeks (range: 2-24 weeks). This figure represents the average number of telelactation sessions per mother rather than a weekly consultation rate (Table 2).

**Table 2:** Feeding Types Before and After Counseling

Feeding Type	Before Counseling (n, %)	After Counseling (n, %)
Mixed fed	27 (62.79%)	16 (37.21%)
Exclusively formula fed	7 (16.28%)	0 (0.00%)
Breastfed with complementary feeding	6 (13.95%)	13 (30.24%)
Exclusively breastfed	3 (6.98%)	14 (32.56%)

**Figure 2:**
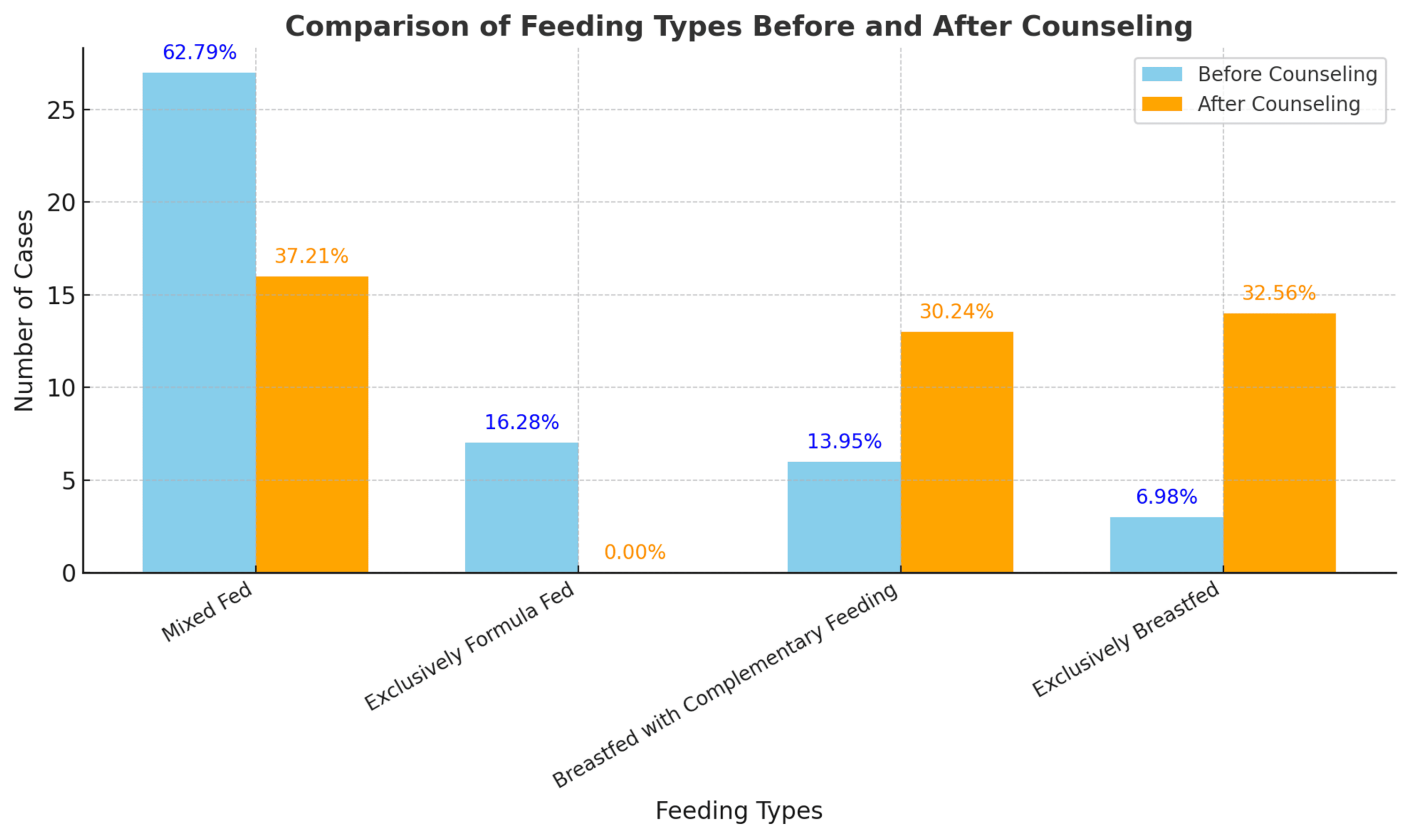
Feeding Types Before and After Counseling

Prior to receiving telelactation counseling, 27 children (62.79%) were mixed-fed with breast milk and formula, seven children (16.28%) were exclusively formula-fed, six children (13.95%) were fed with breast milk and complementary foods, and only three children (6.98%) were exclusively breastfed (Figure 1). Following telelactation counseling, the feeding patterns changed as follows: 16 children (37.21%) continued mixed feeding, 14 children (32.56%) transitioned to exclusive breastfeeding, and 13 children (30.24%) were fed breast milk and complementary foods. Notably, no children remained exclusively formula-fed after counseling (Figure 2, Table 2).

Among the seven mothers undergoing relactation interventions, five (71.42%) successfully re-established exclusive breastfeeding, while two children continued to receive both breast milk and formula. Additionally, one mother with hyperlactation and mastitis successfully regulated her milk production following the cessation of excessive pumping.

During follow-up, the number of infants and young children receiving breast milk and complementary foods increased from 6 to 13, following the implementation of appropriate complementary feeding recommendations after six months of age. One-month post-counseling follow-up interviews indicated that all participating mothers expressed satisfaction with the telelactation support they received.

## Discussion

In emergency situations, supporting mothers is crucial for the continuation of breastfeeding. In our study, telelactation support implemented following the February 2023 earthquake was effective in addressing breastfeeding-related challenges, including relactation, and mothers reported satisfaction with the intervention. Previously, telelactation interventions were primarily used out of necessity, but their application became more widespread during the COVID-19 pandemic [16,22,23].

Due to the fear of infection transmission, the pandemic led to increased mother-infant separation, resulting in a greater reliance on formula feeding. The lack of accessible breastfeeding counseling necessitated the integration of telelactation into clinical guidelines [24]. Additionally, telelactation has emerged as a promising approach in emergency settings, where limited support, concerns about low milk supply, and breastfeeding difficulties are prevalent [22,24]. Beyond providing medical counseling, telelactation also serves as a valuable source of psychological support for mothers, ensuring they do not feel isolated, particularly in situations where preventive healthcare services and social support are lacking [22,25].

Prior to receiving telelactation counseling, 62.79% of infants were mixed-fed with breast milk and formula, 16.28% were exclusively formula-fed, 13.95% were fed with breast milk and complementary foods, and only 6.98% were exclusively breastfed. Following telelactation counseling, exclusive breastfeeding rates increased to 32.56%, while no infants remained exclusively formula-fed. The number of infants receiving breast milk alongside complementary foods increased from 6 to 13, highlighting the role of telelactation in facilitating continued breastfeeding while transitioning to complementary feeding.

Recent studies have demonstrated that tele-breastfeeding is an effective method for supporting breastfeeding. A study conducted by Jerin et al. in Bangladesh found that post-discharge support via mobile phone improved breastfeeding outcomes, while Seyyedi et al. reported similar benefits through mobile application-based breastfeeding interventions [23,24]. Similarly, Cavalcanti et al. found that weekly telelactation support within a closed group increased breastfeeding success rates [25]. In a randomized controlled trial by Uzunçakmak et al., involving 68 participants, mothers who received social media-based breastfeeding counseling showed significantly improved breastfeeding self-efficacy levels [26]. Additionally, a systematic review of 73 randomized controlled trials in the Cochrane database found that breastfeeding initiation rates were significantly higher among groups utilizing telemedicine-based breastfeeding support [27].

Consistent with these findings, our study demonstrated a substantial increase in exclusive breastfeeding rates following telelactation counseling. Additionally, five out of seven mothers (71.42%) undergoing relactation successfully transitioned to exclusive breastfeeding, further underscoring the role of telelactation in supporting breastfeeding continuation.

Although access to telelactation services varies within the same country due to factors such as availability of communication devices, digital literacy, economic and educational disparities, access to healthcare services, and regional healthcare policies, mothers reported that receiving breastfeeding counseling via telelactation, without the need to visit a healthcare facility, was a satisfactory experience [21,23,25].

While telelactation cannot fully replace in-person breastfeeding consultations, it serves as a practical and effective alternative in emergency situations when supported by appropriate technological infrastructure and clinical guidance [22,24].

A key strength of our study is that, to date, it is the first study to evaluate the success of telelactation in a post-disaster setting, excluding research conducted during the COVID-19 pandemic. However, a limitation of our study is the relatively small sample size.

The limited sample size may be attributed to the study being conducted in a post-disaster setting, where participation in telelactation services was inherently restricted. Additionally, as telelactation is still a relatively novel approach compared to traditional in-person breastfeeding counseling, the number of mothers actively seeking this service remained limited. Nonetheless, previous studies evaluating telelactation interventions have also been conducted with small sample sizes, as telelactation-based support is often targeted at specific groups [25,26]. Therefore, despite the sample size constraint, our findings provide valuable insights into the effectiveness of telelactation in disaster contexts and contribute to the growing body of literature on telehealth-based breastfeeding support.

## Conclusions

Telehealth services have a critical role in meeting health needs and maintaining breastfeeding support during disruptions in traditional health systems. Our study provides valuable evidence on the application of telelactation in disaster scenarios, while highlighting its potential to increase breastfeeding rates and improve maternal satisfaction. Although not a substitute for personal care, telelactation has demonstrated practicality and effectiveness as a complementary approach and contributes significantly to the global framework for emergency infant and young child feeding (IYCF-E).
